# Timing for deep vein thrombosis chemoprophylaxis in traumatic brain injury: an evidence-based review

**DOI:** 10.1186/s13054-015-0814-z

**Published:** 2015-03-24

**Authors:** Hiba Abdel-Aziz, C Michael Dunham, Rema J Malik, Barbara M Hileman

**Affiliations:** General Surgery/Trauma Services/Surgical Critical Care, St Elizabeth Health Center, 1044 Belmont Avenue, Youngstown, OH 44501 USA

## Abstract

Multiple studies have addressed deep vein thrombosis chemoprophylaxis timing in traumatic brain injuries. However, a precise time for safe and effective chemoprophylaxis is uncertain according to experts. A comprehensive literature review on brain injuries was performed to delineate temporal proportions for 1) spontaneous intracranial hemorrhage (ICH) progression, 2) post-chemoprophylaxis ICH expansion, and 3) post-chemoprophylaxis deep vein thrombosis. Twenty-three publications were found including more than 5,000 patients. Spontaneous ICH expansion at 24 hours was 14.8% in 1,437 patients from chemoprophylaxis studies and 29.9% in 1,257 patients not in chemoprophylaxis studies (*P* < 0.0001). With low-risk ICH (n = 136), 99% of spontaneous ICH expansion occurred within 48 hours. In moderate or high-risk ICH (n = 109), 18% of spontaneous ICH expansion occurred after day 3. If patients with pre-chemoprophylaxis ICH expansion are included, the post-chemoprophylaxis ICH expansion proportion was 5.6% in 1,258 patients with chemoprophylaxis on days 1 to 3 and was 1.5% in 401 with chemoprophylaxis after day 3 (*P* = 0.0116). If patients with pre-chemoprophylaxis ICH expansion were excluded, the post-chemoprophylaxis ICH expansion proportion was 3.1% in 1,570 patients with chemoprophylaxis on days 1 to 3 and was 2.8% in 582 with chemoprophylaxis after day 3 (*P* = 0.7769). In diffuse axonal injury (n = 188), the post-chemoprophylaxis ICH expansion proportion was 1.6% with chemoprophylaxis after day 3. The deep vein thrombosis proportions were as follows: chemoprophylaxis on days 1 to 3, 2.6% in 2,384 patients; chemoprophylaxis on days 4 or 5, 2.2% in 831; and chemoprophylaxis on day 8, 14.1% in 99 (*P* < 0.0001). Spontaneous ICH expansion proportions at 24 hours substantially vary between chemoprophylaxis and non-chemoprophylaxis studies. Chemoprophylaxis should not be given within 3 days of injury for moderate-risk or high-risk ICH. Chemoprophylaxis is reasonable when low-risk patients have not developed ICH expansion within 48 hours post-injury. Chemoprophylaxis is also acceptable after day 3, when low-risk patients develop ICH expansion within 48 hours post-injury. In diffuse axonal injury patients who have not developed ICH within 72 hours, chemoprophylaxis is reasonable. Deep vein thrombosis proportions significantly increase when chemoprophylaxis is withheld for greater than 7 days.

## Introduction

Multiple publications have addressed the issue of timing of deep vein thrombosis (DVT) chemoprophylaxis in traumatic brain injury (TBI) patients [1]. This literature has been complemented with a 2010 decision analysis, published in *Critical Care* [2]. In 2012, Phelan objectively summarized the primary issues regarding the administration of chemoprophylaxis in TBI patients [1]. This review indicated that there is no accepted standard for the optimal use of chemoprophylaxis in these patients. Of concern is an earlier publication documenting that 54% of TBI patients developed DVT [3]. The primary clinical concern is that the administration of chemoprophylaxis may cause intracranial hemorrhage (ICH) expansion and the potential for neurologic deterioration [1]. A substantial percentage of TBI patients will undergo spontaneous (non-chemoprophylaxis-related) ICH expansion, an observation often related to the magnitude of the initial ICH [4-7]. Phelan described the notion of an early time period when the risk for spontaneous ICH expansion should prohibit chemoprophylaxis administration. He further indicated that while there is likely a later time when the spontaneous and post-chemoprophylaxis ICH expansion risks are minimal, delays in chemoprophylaxis administration may be associated with unacceptably high DVT proportions. Therefore, Phelan suggested that a more qualitative assessment of these risky and safe time points is needed to assist clinicians with appropriate chemoprophylaxis administration. Numerous studies indicated the proclivity of previous investigators to consider ICH as an all-or-none phenomenon; yet, additional literature indicated that the propensity for spontaneous ICH expansion differs according to varying ICH traits. Hence, repeated emphasis is placed on the need for clinicians to understand that the risk for spontaneous ICH expansion is variable, which suggests that the appropriate time for chemoprophylaxis administration should also differ.

The Brain Trauma Foundation suggests that low molecular weight heparin (LMWH) or low-dose unfractionated heparin should be used with mechanical prophylaxis to prevent TBI DVT [8]. However, there is insufficient evidence to support specific recommendations regarding the preferred agent, dose, and timing of pharmacologic prophylaxis for DVT.

Our aim was to review the published literature for evidence that addresses four issues regarding chemoprophylaxis in patients with TBI. The first major objective was to collate post-chemoprophylaxis ICH expansion and DVT proportions according to the post-injury day of chemoprophylaxis administration. Second, we aimed to determine whether unfractionated heparin or LMWH is more efficacious or harmful, compared with the other. Third, we assessed the impact of routine venous surveillance on DVT proportions. Finally, we sought to determine the ubiquity with which intermittent pneumatic compression devices were utilized in relevant TBI cohorts assessing DVT complications.

## Review methods

### Literature search and level of evidence assessment

The initial search spanned a 10-year period (2003 to 2012) and was performed in PubMed using Medical Subject Heading (MeSH) terms. ‘Head injuries’ and ‘Intracranial hemorrhage, traumatic’ were the two primary MeSH categories. Each primary MeSH term was combined with each of the following secondary MeSH terms: ‘Anticoagulants’, ‘Enoxaparin’, ‘Heparin’, ‘Venous thromboembolism’, and ‘Venous thrombosis’. PubMed searches were performed, using all 10 combinations of the primary and secondary MeSH terms. With each interrogation, all potentially relevant review articles and investigations were assessed. When the abstract content suggested possible significance, the manuscript was obtained and reviewed. Manuscripts of investigations were assessed to determine if relevant information existed within the publication. Further, the bibliography of relevant investigations was reviewed to find additional, potentially germane studies. Finally, appropriate review article bibliographies were evaluated to identify additional studies that might contain pertinent information. The level of evidence was classified for each article selected for inclusion in the review [9].

### Spontaneous intracranial hemorrhage expansion investigations

Multiple data results were considered relevant for investigations assessing spontaneous traumatic ICH expansion proportions. These data included the inclusion and exclusion criteria, patient injury traits, and the percentage of the cohort with ICH. Patient injury traits included admission Glasgow Coma Score (GCS), Injury Severity Score, head Abbreviated Injury Score (AIS), the Marshall score, and magnitude of the initial ICH. Spontaneous ICH expansion proportions with timelines were deemed essential. Investigations were stratified and assessed according to whether there was intent to assess for chemoprophylaxis sequelae. ICH proportions were those as described in the results section of each manuscript.

### Post-chemoprophylaxis intracranial hemorrhage expansion proportions

Several data outcomes were considered pertinent for studies assessing post-chemoprophylaxis traumatic ICH expansion proportions. These data included the inclusion and exclusion criteria, patient injury traits, and the percentage of the cohort with ICH. ICH proportions were those as described in the results section of each manuscript. Patient injury traits included admission GCS, Injury Severity Score, head AIS, the Marshall score, and magnitude of the initial ICH. Post-chemoprophylaxis ICH expansion proportions were considered as critical information. Investigations were categorized and evaluated according to whether pre-chemoprophylaxis ICH patients were included or excluded in the cohort analysis. The chemoprophylaxis agents administered were classified as unfractionated heparin, LMWH, or either. The time of chemoprophylaxis administration was deemed to be essential and was documented as a categorical time or, preferably, according to the precise time (hours or days post-injury), if documented.

### Deep vein thrombosis proportions

Because DVT proportions were described for most of the studies investigating post-chemoprophylaxis ICH expansion, these estimates were considered to be the most relevant for the current analysis. The proportion of DVT occurrence, as reported in each results section, was considered as the most essential documented finding. The chemoprophylaxis agents administered were classified as unfractionated heparin, LMWH, or either. The time of chemoprophylaxis administration was documented as a categorical time or, preferably, according to the precise time, when available. Each article describing a DVT proportion was assessed for documentation in the methods section that intermittent pneumatic compression devices were utilized and whether DVT surveillance was routine.

### Statistical analysis

When event proportions from individual studies were combined, the number of patients assessed in each study was summed and the number of patients with an event in each study was totaled. The combined event proportion was the total number of patients with an event divided by the total number of patients under observation. Combined event proportions were compared with other combined event proportions, according to differences in an intervention or an alternative characteristic. Intergroup event proportions were compared using Chi-square or Fisher’s exact testing, as appropriate. Epi Info™ 7.0.9.7 (Centers for Disease Control and Prevention, Atlanta, GA, USA, 2012) was utilized to perform intergroup event proportion statistical analyses.

## Review results

### Literature search and levels of evidence

A summary of the literature search process is described in Table 1. A study by Kwiatt and colleagues [10] was identified as potentially relevant; however, it was not included in the analysis because 20% of the patients presented in earlier reports were included in our analysis. The review includes 23 studies, with the following levels of evidence: 11 level 3 studies, 6 level 4 studies, and 6 level 5 studies (Table 2).

**Table 1:** **PRISMA 2009 flow table**

	**Number**	**Reasons**
Records identified through PubMed	595	
Records after duplicates removed	321	
Records screened	321	
Full-text articles assessed for eligibility	44	
Full-text articles excluded	20	No pertinent data
Studies included in quantitative synthesis	23

**Table 2:** **Level of evidence for studies included in the literature review**

**Study**	**Prospective**	**Comparison group**	**Level of evidence**
**CP studies**			
Arnold *et al.* [28]	No	Yes	3
Cothren *et al.* [21]	Yes	No	5
Depew *et al.* [22]	No	Yes	4
Dudley *et al.* [17]	No	Yes	4
Kim *et al.* [18]	No	Yes	4
Kleindienst *et al.* [23]	No	No	5
Koehler *et al.* [19]	No	Yes	4
Kurtoglu *et al.* [20]	Yes	Yes	3
Levy *et al.* [11]	No	Yes	3
Minshall *et al.* [12]	No	Yes	3
Norwood *et al.* [24]	Yes	No	5
Norwood *et al.* [13]	Yes	No	5
Norwood *et al.* [14]	Yes	No	5
Pahatouridis *et al.* [25]	Yes	No	5
Phelan *et al.* [26]	Yes	Yes	3
Saadeh *et al.* [15]	No	Yes	4
Salottolo *et al.* [27]	No	Yes	3
Scudday *et al.* [29]	No	Yes	3
**Non-CP studies**			
Bee *et al.* [4]	No	Yes	4
Chang *et al.* [5]	No	Yes	3
Park *et al.* [6]	No	Yes	3
Phelan [16]	Yes	Yes	3
Velmahos *et al.* [7]	No	Yes	3

CP, chemoprophylaxis.

### Spontaneous intracranial hemorrhage expansion proportions

Spontaneous ICH expansion proportions come from studies investigating DVT chemoprophylaxis outcomes and from other investigations where there was no intent to assess chemoprophylaxis sequelae. Of the five chemoprophylaxis cohorts, spontaneous ICH expansion at 24 hours was 14.8% and included 1,437 patients [11-15]. In the investigations without intent to assess the impact of chemoprophylaxis, spontaneous ICH expansion at 24 hours was 29.9% in eight cohorts that included 1,257 patients [4-7,16]. The spontaneous ICH expansion proportion was significantly different (*P* < 0.0001; odds ratio 2.5 (95% confidence interval (CI) 2.0 to 3.0). Of the five chemoprophylaxis cohorts, the initial ICH proportion was ≤50% in one study [11], not documented in another investigation [14], approximately 85% in a third study [12], and 100% in the remaining two studies [13,15]. For the three studies that documented an ICH ≥85% on the initial computed tomography (CT) scan, the ICH expansion proportion at 24 hours was 13.5% (77/572) [12,13,15]. For the non- chemoprophylaxis studies, virtually all patients initially had an ICH.

Spontaneous ICH expansion proportions, with delineated timelines, were presented in a publication by Phelan and colleagues [16]. Low-risk ICH traits were epidural or subdural hematoma <9 mm, cerebral contusion <2 cm, single contusion per lobe, traumatic subarachnoid hemorrhage with a negative CT angiography, and intraventricular hemorrhage <2 cm in maximum diameter. Moderate-risk ICH was an ICH that was a greater degree of hemorrhage than that described for the low-risk criteria. High-risk ICH included patients who required a craniotomy or intracranial pressure monitoring. Of the 136 with low-risk ICH, ICH expansion occurred in 34 (25.0%), where the ICH expansion was at its largest size by 48 hours post-injury in 98.5%. Of the 42 with moderate-risk ICH, ICH expansion occurred in 18 (42.9%), where ICH expansion was not at its largest size until after 72 hours post-injury in 22.2% (4/18) (95% CI 9.0 to 45.2%). Of the 67 with high-risk ICH, ICH expansion occurred in 43 (64.2%), where the ICH expansion was not at its maximal size until after 72 hours post-injury in 16.3% (7/43) (95% CI 8.1 to 30.0%).

### Post-chemoprophylaxis intracranial hemorrhage expansion proportions

The comprehensive literature search found 26 cohorts in 18 studies (4,005 patients) that described ICH expansion proportions following chemoprophylaxis. Table 3 summarizes select traits for patients in studies that received chemoprophylaxis and included some patients with pre-chemoprophylaxis ICH expansion. The table includes: the percentage of patients with ICH, when known; admission GCS, head AIS or Injury Severity Score; reasons for study exclusion; and initiation time for chemoprophylaxis, when relevant. Table 4 summarizes specified traits for patients in studies that received chemoprophylaxis and excluded all patients with pre-chemoprophylaxis ICH expansion. The table includes: the percentage of patients with ICH, when known; admission GCS, head AIS or Marshall score; other reasons for study exclusion; and initiation time for chemoprophylaxis, when relevant. Table 5 delineates select study characteristics and outcomes: 1) whether patients with pre-chemoprophylaxis ICH expansion were included or excluded; 2) chemoprophylaxis agent; 3) day of chemoprophylaxis initiation; 4) cohort size; and 5) proportion of post-chemoprophylaxis ICH expansion. For studies that included some patients with pre-chemoprophylaxis ICH expansion, the post-chemoprophylaxis ICH proportion was 5.6% (70/1,258) when chemoprophylaxis was given on post-injury days 1 to 3 [11,12,15,17-20]. A single study, which included some patients with pre-chemoprophylaxis ICH expansion, showed that the post-chemoprophylaxis ICH proportion was 1.5% (6/401) when chemoprophylaxis was given after post-injury day 3 [19]. The proportion of difference for chemoprophylaxis at days 1 to 3 versus after day 3 was significant (*P* = 0.0116; odds ratio = 3.9 (95% CI 1.6 to 9.0%)).

**Table 3:** **Patient traits for chemoprophylaxis studies that included those with early increased intracranial hemorrhage**

**Study**	**ICH percentage**	**GCS/hAIS**	**Exclusions**	**CP day**
Dudley *et al.* [17]	NR	GCS 7	None relevant	3
Kim *et al.* [18]	100	GCS 9, hAIS >3	Very few patients excluded	≤3
Koehler *et al.* [19]	100	hAIS 3.7	ICP device	3
Koehler *et al.* [19]	100	hAIS 3.9	ICP device	5
Kurtoglu *et al.* [20]	90	GCS 3-8	Craniotomy	1
Levy *et al.* [11]	~50	hAIS 4	Hospital LOS <3 days	3
Minshall *et al.* [12]	85	hAIS 3.8	Hospital LOS ≤48 hours	47 hours
Minshall *et al.* [12]	85	hAIS 4.1	Hospital LOS ≤48 hours	55 hours
Saadeh *et al.* [15]	100	NR	Hospital LOS <3 days; no repeat CT	2
Saadeh *et al.* [15]	100	NR	Hospital LOS <3 days; no repeat CT	≥3

CP, chemoprophylaxis; CT, computed tomography; GCS, Glasgow Coma Score; hAIS, head Abbreviated Injury Scale score; ICH, intracranial hemorrhage; ICP, intracranial pressure; LOS, length of stay; NR, not reported.

**Table 4:** **Patient traits for chemoprophylaxis studies that excluded those with early increased intracranial hemorrhage**

**Study**	**ICH percentage**	**GCS/hAIS**	**Other exclusions**	**CP day**
Arnold *et al.* [28]	100	NR	DAI; cerebral edema; craniotomy	-
Cothren *et al.* [21]	NR	GCS 3-7	systemic anticoagulation; diagnosis of DVT; placement of vena cava filter	-
Depew *et al.* [22]	100	MS ≥2	Mass-effect	-
Kleindienst *et al.* [23]	36	NR	None; 16% of candidates excluded	-
Levy *et al.* [11]	~50	hAIS 4	Hospital LOS <3 days	3
Norwood *et al.* [24]	NR	GCS 3-8	LOS <3 days; ISS <9; spinal cord injury; coagulopathy; LMWH not at appropriate time; no duplex scan at discharge; high risk for bleeding	-
Norwood *et al.* [24]	NR	hAIS ≥2	See above	-
Norwood *et al.* [13]	100	GCS 10.0, hAIS 3.6	LOS <2 days; coagulopathy; expected brain death	-
Norwood *et al.* [14]	NR	GCS 10.4, hAIS 3.6	Surgeon reluctance despite meeting criteria n = 24%; large ICH; persistent ICP >20; expected brain death; hospital LOS <3 days; solid organ injury; spinal cord hematoma; coagulopathy; pre-injury antithrombotic	-
Pahatouridis *et al.* [25]	NR	GCS 9-12	Extra-cranial injury; surgery; coagulopathy	-
Phelan *et al.* [26]	96	GCS 13.5	Large ICH; persistent ICP >20 torr	1
Phelan *et al.* [26]	93	GCS 13.0	Large ICH; persistent ICP >20 torr	4
Salottolo *et al.* [27]	NR	GCS ≤8 (29%), hAIS 3.5	Hospital LOS <3 days; death in 7 days; IVC filter; pre-injury antithrombotic	-
Scudday *et al.* [29]	NR	GCS >9 (50%), hAIS 3.4	Craniotomy; hospital LOS ≤3 days	-

Dashes in the ‘CP day’ column indicate no ICH percentage, GCS, hAIS, or patient exclusion variance according to day of CP administration. CP, chemoprophylaxis; DAI, diffuse axonal injury; DVT, deep vein thrombosis; GCS, Glasgow Coma Score; hAIS, head Abbreviated Injury Scale score; ICP, intracranial pressure; ICH, intracranial hemorrhage; ISS, Injury Severity Score; IVC, inferior vena cava; LOS, length of stay; LMWH, low molecular weight heparin; MS, Marshall score; NR, not reported.

**Table 5:** **Post-chemoprophylaxis intracranial hemorrhage expansion**

**Study**	**Early ↑ ICH**	**Agent**	**CP day**	**Number**	**ICH ↑**	**Percentage**
Arnold *et al.* [28]	No	E	8	99	2	2.0
Cothren *et al.* [21]	No	L	3	174	0	0.0
Depew *et al.* [22]	No	E	≤2	29	1	3.5
Depew *et al.* [22]	No	E	>3	53	2	3.8
Dudley *et al.* [17]	Yes	L	3	287	1	0.3
Kim *et al.* [18]	Yes	H	≤3	47	0	0.0
Kleindienst *et al.* [23]	No	L	1	271	0	0.0
Koehler *et al.* [19]	Yes	L	3	268	4	1.5
Koehler *et al.* [19]	Yes	L	5	401	6	1.5
Kurtoglu *et al.* [20]	Yes	L	1	60	1	1.7
Levy *et al.* [11]	Yes	L	3	221	36	16.3
Levy *et al.* [11]	No	L	3	163	15	9.2
Minshall *et al.* [12]	Yes	L	2	158	8	5.1
Minshall *et al.* [12]	Yes	H	2	171	20	11.7
Norwood *et al.* [24]	No	L	1	36	0	0.0
Norwood *et al.* [24]	No	L	1	19	0	0.0
Norwood *et al.* [13]	No	L	1	150	6	4.0
Norwood *et al.* [14]	No	L	2	525	18	3.4
Pahatouridis *et al.* [25]	No	L	1	61	0	0.0
Phelan *et al.* [26]	No	L	1	34	2	5.9
Phelan *et al.* [26]	No	L	4	28	1	3.6
Saadeh *et al.* [15]	Yes	L	2	46	0	0.0
Saadeh *et al.* [15]	Yes	L	≥3	47	0	0.0
Salottolo *et al.* [27]	No	L	<3	108	7	6.5
Salottolo *et al.* [27]	No	L	≥3	147	21	14.3
Scudday *et al.* [29]	No	E	4	402	11	2.7
Total				4,005	162	4.0

Early ↑ ICH, included patients with pre-chemoprophylaxis intracranial hemorrhage (ICH) expansion; ↑ ICH, post-CP intracranial hemorrhage expansion; CP, chemoprophylaxis; E, either unfractionated heparin or low molecular weight heparin; H, unfractionated heparin; L, low molecular weight heparin.

For studies that excluded all patients with pre-chemoprophylaxis ICH expansion, the post-chemoprophylaxis ICH proportion was 3.1% (49/1,570) when chemoprophylaxis was given on post-injury days 1 to 3 [11,13,14,21-27]. For studies that excluded all patients with pre-chemoprophylaxis ICH expansion, the post-chemoprophylaxis ICH proportion was 2.8% (16/582) when chemoprophylaxis was given after post-injury day 3 [22,26,28,29]. The proportion difference for chemoprophylaxis at days 1 to 3 versus after day 3 was not significant (*P* = 0.7769). One study investigating diffuse axonal injury (n = 118) found that any ICH expansion occurred within 72 hours post-injury [19]. When chemoprophylaxis was given at day 4, the investigators found that the post-chemoprophylaxis ICH expansion proportion was 1.6%. Post-chemoprophylaxis ICH expansion was greater with unfractionated heparin (9.2%; 20/218) [12,18] compared with LMWH (3.9%; 126/3,204) (*P* = 0.0008) [11-15,17,19-21,23-27].

### Deep vein thrombosis proportions

Because DVT proportions were described for most of the studies investigating post-chemoprophylaxis ICH expansion, these estimates were the most relevant evidence. These comprised 28 cohorts in 15 studies (4,491 patients; Table 6). Table 6 delineates select study characteristics: 1) chemoprophylaxis agent used (unfractionated heparin, LMWH, or either); 2) lower extremity compression device use; 3) chemoprophylaxis day of administration; 4) cohort size; and 5) DVT proportion. Intermittent pneumatic compression devices were used in 78.6% (22/28) of the cohorts and one cohort used compression stockings (Table 6). However, there was no statement regarding lower extremity compression devices for the other five cohorts (Table 6). Table 7 delineates the DVT proportions for patients where 1) chemoprophylaxis was never considered appropriate [11,12,20,22,27,29], 2) chemoprophylaxis was given on post-injury days 1 to 3 [12-15,17-20,22,23,26,27], 3) chemoprophylaxis was given on post-injury day 4 or 5 [19,26,29], and 4) chemoprophylaxis was given on post-injury day 8 [28]. The DVT proportion for chemoprophylaxis on day 8 was significantly higher than for the other groups (*P* < 0.0001). The DVT proportion for patients receiving unfractionated heparin was 4.6% (13/282) [12,18,28], whereas the DVT proportion for patients receiving LMWH was 2.8% (79/2,812) (Table 6) [11-15,17,19,20,23,26-28]. The DVT proportion with unfractionated heparin (4.6%) was not statistically different from the LMWH proportion (2.8%; *P* = 0.0968). For patients receiving chemoprophylaxis and undergoing routine DVT scanning, the DVT proportion was 5.4% (37/682; Table 6) [11,18,20,22,27]. For patients receiving chemoprophylaxis and not undergoing routine DVT scanning, the DVT proportion was 2.3% (68/2,896; Table 6) [12-15,17,19,23,26,28,29]. The DVT proportion was significantly higher in patients undergoing routine DVT surveillance scanning (relative risk 2.3 (95% CI 1.6 to 34); *P* < 0.0001).

**Table 6:** **Deep vein thrombosis proportions**

**Study**	**Agent**	**IPC**	**IPCdur**	**CP day**	**RS**	**Number**	**DVT**	**Percentage**
Arnold *et al.* [28]	H	Yes	NS	8	No	47	8	17.0
Arnold *et al.* [28]	L	Yes	NS	8	No	52	6	11.5
Depew *et al.* [22]	E	Yes	Amb	<3	Yes	29	4	13.8
Depew *et al.* [22]	E	Yes	Amb	>3	Yes	53	6	11.3
Depew *et al.* [22]	N	Yes	Amb	None	??	42	0	0.0
Dudley *et al.* [17]	L	Yes	Amb	3	No	287	21	7.3
Kim *et al.* [18]	H	Yes	Amb	≤3	Yes	47	2	4.3
Kim *et al.* [18]	H	Yes	Amb	>3	Yes	17	1	5.9
Kleindienst *et al.* [23]	L	CS	Amb	1	No	280	0	0.0
Koehler *et al.* [19]	L	NS	NS	3	No	268	4	1.5
Koehler *et al.* [19]	L	NS	NS	5	No	401	14	3.5
Kurtoglu *et al.* [20]	L	Yes	NS	1	Yes	60	3	5.0
Kurtoglu *et al.* [20]	N	Yes	NS	None	Yes	60	4	6.7
Levy *et al.* [11]	L	Yes	Amb	3	Yes	221	13	5.9
Levy *et al.* [11]	N	Yes	Amb	None	Yes	119	2	1.7
Minshall *et al.* [12]	N	Yes	NS	None	No	57	1	1.8
Minshall *et al.* [12]	L	Yes	NS	2	No	158	1	0.6
Minshall *et al.* [12]	H	Yes	NS	2	No	171	2	1.2
Norwood *et al.* [13]	L	Yes	CP	1	No	150	2	1.3
Norwood *et al.* [14]	L	NS	NS	2	No	525	6	1.1
Phelan *et al.* [26]	L	NS	NS	1	No	34	0	0.0
Phelan *et al.* [26]	L	NS	NS	4	No	28	1	3.6
Saadeh *et al.* [15]	L	Yes	NS	2	No	46	0	0.0
Saadeh *et al.* [15]	L	Yes	NS	≥3	No	47	0	0.0
Salottolo *et al.* [27]	N	Yes	NS	None	Yes	225	4	1.8
Salottolo *et al.* [27]	L	Yes	NS	<3	Yes	108	5	4.6
Salottolo *et al.* [27]	L	Yes	NS	≥3	Yes	147	3	2.0
Scudday *et al.* [29]	E	Yes	NS	4	No	402	3	0.8
Scudday *et al.* [29]	N	Yes	NS	None	Yes	410	11	2.7
Total						4,491	127	2.8

Amb, until ambulating; CP, chemoprophylaxis; CS, compression stockings; DVT, deep vein thrombosis ; E, either unfractionated heparin or low molecular weight heparin; H, unfractionated heparin; IPC, intermittent pneumatic compression devices; IPCdur, intermittent pneumatic compression duration; L, low molecular weight heparin; NS, not stated; RS, routine deep vein thrombosis surveillance.

**Table 7:** **Deep vein thrombosis proportions according to chemoprophylaxis timing**

**CP day**	**Number**	**DVT**	**Percentage**	**95% CI**
Not given	913	22	2.4%	1.5-3.6%
Days 1 to 3	2,384	63	2.6%	2.1-3.4%
Days 4 or 5	831	18	2.2%	1.4-3.4%
Day 8	99	14	14.1%	8.6-22.4%

CP, chemoprophylaxis; CI, confidence interval; DVT, deep vein thrombosis.

## Discussion

### Spontaneous intracranial hemorrhage expansion at 24 hours

Spontaneous ICH expansion proportions at 24 hours come from studies investigating chemoprophylaxis outcomes and from other investigations where there was no intent to assess chemoprophylaxis sequelae. The ICH expansion proportion at 24 hours in investigations without the intent to assess the impact of chemoprophylaxis was twice that of studies directed at evaluating post-chemoprophylaxis ICH expansion. Virtually all patients in studies without the intent to assess the impact of chemoprophylaxis on ICH expansion had ICH on the initial CT scan. In contrast, the initial ICH proportion for the chemoprophylaxis studies was commonly <100% or not documented. In the three chemoprophylaxis studies with an initial ICH proportion of 85 to 100%, the ICH expansion proportion at 24 hours was substantially less, compared with the studies without the intent to assess chemoprophylaxis sequelae. This suggests that the patient cohort traits for the two groups of studies are at variance; specifically, patients undergoing pre-chemoprophylaxis analysis were likely biased by the various inclusion and exclusion criteria used.

### Spontaneous intracranial hemorrhage expansion proportions with delineated timelines

Phelan and colleagues provided insight into spontaneous ICH progression without bias from multiple exclusion criteria [16]. Virtually all patients with low-risk ICH had suffered spontaneous ICH expansion at 48 hours post-injury. Conversely, a substantial portion of patients with moderate or high-risk ICH developed ICH expansion >72 hours post-injury. These findings suggest that chemoprophylaxis would be reasonable in low-risk ICH patients with a stable brain CT at 48 hours. However, in those with moderate or high risk ICH, chemoprophylaxis would not be appropriate until >72 hours post-injury.

### Post-chemoprophylaxis intracranial hemorrhage expansion proportions

The comprehensive literature search found investigations describing ICH expansion proportions following chemoprophylaxis for 4,000 patients. For the 10 cohorts described in seven studies that included some patients with pre-chemoprophylaxis ICH expansion, the post-chemoprophylaxis ICH proportion was significantly greater when chemoprophylaxis was given on post-injury days 1 to 3 (5.6%) compared with chemoprophylaxis given after day 3 (1.5%). Therefore, chemoprophylaxis during the 72 hours post-injury was associated with a risk of post-chemoprophylaxis ICH expansion in patients with spontaneous pre-chemoprophylaxis ICH expansion. The pre-chemoprophylaxis ICH proportion ranged from 85 to 100% in 8 of the 10 study cohorts investigated; however, the proportion was not documented or was <85% in the other two cohorts. Most of the studies excluded very few critical patients; alternatively, one study [19] excluded patients requiring an intracranial pressure device and another investigation [20] excluded those requiring craniotomy. This implies that chemoprophylaxis during the first 72 hours increases the risk for post-chemoprophylaxis expansion in patients with ICH on the initial CT, especially when there has been pre-chemoprophylaxis ICH expansion.

The investigations that excluded patients with pre chemoprophylaxis ICH expansion indicated that the post-chemoprophylaxis ICH proportion was similar when chemoprophylaxis was given on post-injury days 1 to 3 (3.1%) compared with chemoprophylaxis given after day 3 (2.8%). Of the 14 relevant cohorts in 12 studies, the initial CT ICH proportion ranged from 93 to 100% in five cohorts and <80% in two cohorts; these data were not stipulated in the other seven cohorts. Within the 14 cohorts, GCS documentation noted severe brain injury in two and non-severe brain injury in seven; the GCS was not documented in five. Half of the cohorts excluded patients with large, complex ICH [13,14,22,26,28,29]. Overall, these findings suggest that chemoprophylaxis during the 72 hours post-injury is unlikely to cause post-chemoprophylaxis ICH expansion when patients with complex ICH or spontaneous pre-chemoprophylaxis ICH expansion are excluded.

An investigation of diffuse axonal injury showed that ICH expansion, if it were to occur, would develop within 72 hours post-injury [19]. Chemoprophylaxis given at post-injury day 4 had a subsequent ICH expansion proportion that was negligible, implying that chemoprophylaxis is reasonable for diffuse axonal injury on day 4. Still, chemoprophylaxis timing should be customized for those with ICH expansion.

When compared with LMWH, post-chemoprophylaxis ICH expansion was greater with unfractionated heparin. This suggests that LMWH is preferable in TBI patients.

### Deep vein thrombosis proportions

We considered the DVT proportions cited in the post-chemoprophylaxis ICH expansion studies to be the most germane to our literature review. Collectively, the publications describe a DVT proportion experience for over 4,000 patients. In the methodology section, most of the studies indicated that intermittent pneumatic compression devices were used, implying that the majority of the patients had those devices applied. The DVT proportions were 2.4% for patients where chemoprophylaxis was inappropriate, 2.6% with chemoprophylaxis given on post-injury days 1 to 3, and 3.4% with chemoprophylaxis given after post-injury day 3. Since these proportions are neither statistically nor clinically different, we can infer that a delay in chemoprophylaxis administration until after post-injury day 3 is not detrimental. Conversely, Arnold and colleagues [28] indicated that a delay in chemoprophylaxis administration until post-injury day 8 is associated with an increase in DVT. Statements in the discussion section indicate that the authors have revised their practice to provide earlier chemoprophylaxis, following review of their data results and recent relevant literature. Delays in chemoprophylaxis during the study period likely represented an institutional perception that earlier administration was risky in patients with ICH and without substantial value.

The DVT proportions for patients receiving unfractionated heparin or LMWH were similar, implying that neither drug is more or less efficacious. DVT proportions were higher in studies utilizing a routine DVT surveillance process compared with DVT assessment only for patients with clinical manifestations.

Because the 54% head injury DVT proportion reported by Geerts and colleagues is at odds with the current review, certain study features are worth elucidating [3]. Initially 716 trauma patient admissions with an Injury Severity Score ≥9 were screened, but half of those were excluded because they did not undergo contrast venography, or the venogram was inadequate (n = 367). Thus, they described the results of 349 patients who had a good-quality lower-extremity contrast venogram 14 to 21 days after admission or earlier if the hospital stay was <14 days. All patients had impedance plethysmography performed every other day and daily clinical surveillance for DVT. Of the 349 patients, 91 (26.1%) had a major head injury. Although these study features indicate an admirable and concerted effort to comprehensively define accurate DVT proportions, certain caveats are worth noting. First, the methodology clearly states that patients did not receive mechanical or pharmacologic antithrombotic prophylaxis during the study. Second, only 1.5% (3/201) of all trauma patients with a DVT by venography had clinical signs of DVT. Third, although the head injury patients had a 54% overall DVT proportion, the incidence of proximal DVT was substantially lower at 19.8% (18/91). Fourth, when compared with the final study group (n = 349), the 367 patients originally excluded from the study were younger, had less severe injuries, and were less likely to have injuries predictive of DVT. Geerts and colleagues’ study, in concert with the current literature review, suggests that head injury intermittent pneumatic compression devices are effective in mitigating DVT and lengthy delays in administering chemoprophylaxis increases DVT proportions. Lastly, both investigations indicate that DVT proportions are likely to increase when routine DVT surveillance is used.

### Complementary review

Of importance is a recent systematic literature review to assess effectiveness and safety of pharmacologic and mechanical prophylaxis, and the optimal time to initiate pharmacologic prophylaxis in TBI [30]. The authors found that evidence existed that enoxaparin reduced rates of DVT and unfractionated heparin reduced rates of mortality compared with no chemoprophylaxis in TBI. They found that the evidence was insufficient to comment on the effectiveness of chemoprophylaxis for DVT started <72 hours versus >72 hours; however, absolute reported proportions were similar. The review indicated that there was insufficient evidence to comment on the effectiveness and safety of mechanical strategies on venothromboembolism outcomes. We believe that Chelladurai and colleagues’ literature review [30] and the current systematic literature synthesis should be considered as complementary efforts.

### Study limitations

There are several limitations in many of the studies used in this analysis. An adequate assessment of TBI severity (that is, GCS, head AIS, and percentage with ICH) was not always available to determine whether groups undergoing one intervention or another were matched for severity of illness. The method used for detecting ICH progression was not always clear; therefore, differences may exist depending on whether only radiology reports were used or scans were re-reviewed by a dedicated neuroradiologist, based on specific *a priori* criteria. Certain studies were biased because attending physicians excluded some patients because of their concern for ICH expansion with early chemoprophylaxis. DVT proportions may have been underestimated because lower extremity ultrasound is unable to interrogate the pelvic veins and the procedure was often not routine. Some studies that failed to demonstrate intergroup differences were underpowered, representing a potential type II error regarding chemoprophylaxis risks or benefits. Therefore, a large randomized, multicenter trial of TBI patients is encouraged to enhance practice management guidelines. Patients should be matched for severity of illness, brain CT and DVT monitoring should be routine, patient selection criteria should be defined, and an investigation methodology should be organized to delineate effective chemoprophylaxis drugs, timing, and doses.

## Conclusion

The spontaneous ICH expansion proportion at 24 hours for investigations without the intent to assess the impact of chemoprophylaxis was twice that of studies directed at evaluating post-chemoprophylaxis ICH expansion. Thus, patients undergoing pre-chemoprophylaxis analysis are likely biased by the various inclusion and exclusion criteria used in those studies. According to the literature, chemoprophylaxis during the first 72 hours is not appropriate for patients with spontaneous ICH expansion or for patients with moderate- or high-risk ICH. There is also evidence that chemoprophylaxis after 48 hours post-injury is unlikely to cause post-chemoprophylaxis ICH expansion when patients with complex ICH or spontaneous pre-chemoprophylaxis ICH expansion are excluded. In patients with diffuse axonal injury, while the literature implies that DVT chemoprophylaxis is reasonable on day 4, chemoprophylaxis timing should be customized for those with ICH expansion. Lower ICH expansion with LMWH indicates that it is preferred over unfractionated heparin for chemoprophylaxis in TBI patients. Furthermore, the evidence implies that DVT proportions do not increase when chemoprophylaxis administration is delayed until post-injury day 4 or 5; however, the proportion significantly increases after 7 days. The literature review suggests that intermittent pneumatic compression devices are effective in mitigating DVT. In addition, neither unfractionated heparin nor LMWH is more or less efficacious for preventing DVT. Finally, the review indicates that DVT proportions are increased when routine surveillance techniques are used.

## Abbreviations

AIS: Abbreviated injury score
CI: Confidence interval
CT: Computed tomography
DVT: Deep vein thrombosis
GCS: Glasgow coma score
ICH: Intracranial hemorrhage
LMWH: Low molecular weight heparin
MeSH: Medical subject heading
TBI: Traumatic brain injury
